# Remdesivir plus Dexamethasone in COVID-19: A cohort study of severe patients requiring high flow oxygen therapy or non-invasive ventilation

**DOI:** 10.1371/journal.pone.0267038

**Published:** 2022-04-28

**Authors:** Gennaro De Pascale, Salvatore Lucio Cutuli, Simone Carelli, Rikardo Xhemalaj, Tommaso Rosà, Giuseppe Bello, Joel Vargas, Melania Cesarano, Luca Montini, Eloisa Sofia Tanzarella, Gabriele Pintaudi, Mariangela Di Muro, Domenico Luca Grieco, Massimo Antonelli

**Affiliations:** 1 Dipartimento Di Scienze Dell’Emergenza, Anestesiologiche e della Rianimazione, Fondazione Policlinico Universitario A. Gemelli IRCCS, Rome, Italy; 2 Istituto di Anestesiologia e Rianimazione, Università Cattolica del Sacro Cuore, Rome, Italy; National and Kapodistrian University of Athens, GREECE

## Abstract

**Introduction:**

Remdesivir and Dexamethasone represent the cornerstone of therapy for critically ill patients with acute hypoxemic respiratory failure caused by Coronavirus Disease 2019 (COVID-19). However, clinical efficacy and safety of concomitant administration of Remdesivir and Dexamethasone (Rem-Dexa) in severe COVID-19 patients on high flow oxygen therapy (HFOT) or non-invasive ventilation (NIV) remains unknown.

**Materials and methods:**

Prospective cohort study that was performed in two medical Intensive Care Units (ICUs) of a tertiary university hospital. The clinical impact of Rem-Dexa administration in hypoxemic patients with COVID-19, who required NIV or HFOT and selected on the simplified acute physiology score II, the sequential organ failure assessment score and the Charlson Comorbidity Index score, was investigated. The primary outcome was 28-day intubation rate; secondary outcomes were end-of-treatment clinical improvement and PaO2/FiO2 ratio, laboratory abnormalities and clinical complications, ICU and hospital length of stay, 28-day and 90-day mortality.

**Results:**

We included 132 patients and found that 28-day intubation rate was significantly lower among Rem-Dexa group (19.7% vs 48.5%, *p*<0.01). Although the end-of-treatment clinical improvement was larger among Rem-Dexa group (69.7% vs 51.5%, *p* = 0.05), the 28-day and 90-day mortalities were similar (4.5% and 10.6% vs. 15.2% and 16.7%; *p* = 0.08 and *p* = 0.45, respectively). The logistic regression and Cox-regression models showed that concomitant Rem-Dexa therapy was associated with a reduction of 28-day intubation rate (OR 0.22, CI95% 0.05–0.94, p = 0.04), in absence of laboratory abnormalities and clinical complications (*p* = ns).

**Conclusions:**

In COVID-19 critically ill patients receiving HFO or NIV, 28-day intubation rate was lower in patients who received Rem-Dexa and this finding corresponded to lower end-of-treatment clinical improvement. The individual contribution of either Remdesevir or Dexamethasone to the observed clinical effect should be further investigated.

## Introduction

Acute respiratory failure (ARF) and acute respiratory distress syndrome (ARDS) are the most frequent life-threatening complications of Coronavirus Disease 2019 (COVID-19) [1, 2]. Respiratory support remains the cornerstone of treatment, with high flow nasal oxygen therapy (HFOT) and non-invasive mechanical ventilation (NIV) widely used as first line therapies in hypoxemic patients [3, 4]. Great interest has been moved toward specific pharmacological therapies to prevent the escalation of respiratory support in patients with COVID-19 related ARF and to improve their overall clinical outcome. The role of corticosteroids in ARDS has been largely studied over time, due to their immunomodulating properties [5–7]. Recently, the RECOVERY trial observed that 10-day therapy with Dexamethasone reduced 28-day mortality in hospitalized COVID-19 patients requiring oxygen support, even in those undergoing invasive mechanical ventilation [8]. Among antiviral agents tested in this setting [9, 10], Remdesivir, a nucleotide prodrug of an adenosine analogue witch binds to the viral RNA-dependent RNA polymerase, has demonstrated in vitro activity against severe acute respiratory syndrome coronavirus 2 (SARS-CoV-2) [11]. *Beigel et al*. randomized a heterogeneous population of patients with COVID-19 related pneumonia, the majority of them requiring no or low flow oxygen therapies, to receive a 10-day therapy with either intravenous Remdesivir or placebo: patients who received Remdesivir had a reduced time to hospital recovery and were more likely to improve their clinical status at day 15 [12]. Conversely, in COVID-19 patients with pneumonia, mainly receiving low flow oxygen support, no significant difference in clinical outcomes was detected between five-day and ten-day courses of therapy with Remdesivir [13].

Thus, a combination therapy with an antiviral and an anti-inflammatory agent may find a rationale in damping the potentially injurious inflammatory response through its etiologic and immunomodulating effects [14]. Although current treatment guidelines for COVID-19 related ARF, in patients requiring low flow oxygen, include the combination of Remdesivir plus Dexamethasone as a therapeutic option [15], clinical data about their concomitant use in patients undergoing HFO/NIV are still lacking.

Hence, our aim was to investigate the efficacy and safety of concomitant Remdesivir and Dexametasone (Rem-Dexa) therapy in COVID-19 critically ill patients admitted to the intensive care unit (ICU) and undergoing high flow nasal oxygen or non-invasive mechanical ventilation.

## Materials and methods

### Study setting and design

This observational study prospectively included hospitalized patients with COVID-19 across two medical ICUs of the Fondazione Policlinico Universitario “A. Gemelli IRCCS” (Rome, Italy), between 1 March 2020 and 15 December 2020, undergoing NIV or HFOT at least ≥ 48 h and receiving a full-course of Dexamethasone (6 mg/iv die for 10 days) plus Remdesivir (200 mg loading dose, then 100 mg/die for a total of five days) (Rem-Dexa group). Electronic patient records and microbiology laboratory data were used to identify patients and to retrieve clinical data (e.g., presence of one or more comorbidities), microbiological results (i.e., from cultures of bronchoalveolar lavage (BAL) fluid or other relevant samples), receipt and/or type of antimicrobial treatment(s), and outcomes. A comparison between patients who received Remdesivir and Dexamethasone (second wave) with those ones who were not treated with such combination (first wave) was made, according to Simplified Acute Physiology Score II (SAPS II) [16], the Sequential Organ Failure Assessment (SOFA) score [17] and the Charlson Comorbidity Index (CCI) [18]. No patients during first wave were readmitted in the second wave. Only patients with less than 10% of missing data were included.

The study was performed in accordance with the Declaration of Helsinki and was approved by the Ethics Committee of the Fondazione Policlinico “A. Gemelli IRCCS” (reference number ID3141). A written informed consent or proxy consent was waived, due to observational nature of the study, according to committee recommendations. All data were anonymous and identified with an admission code number.

### Definitions and outcomes

Oxygen-support status was assessed by a seven-point ordinal scale, as follows: 1, death; 2, receiving invasive mechanical ventilation; 3, receiving high-flow oxygen or non-invasive ventilation; 4, receiving low-flow oxygen; 5 or 6, breathing ambient air; and 7, discharge. End-of-treatment (EOT) clinical improvement was defined as an improvement of at least 1 point from the baseline score of 3 (high-flow oxygenation or non-invasive ventilation) [13]. Clinical outcomes were independently evaluated by two physicians (GDP, RX) who were blinded to the treatment. When judgments were discordant (< 5% of patients), the reviewers reassessed the data and reached a consensus decision. Comparison of oxygen support, according to Rem-Dexa treatment, was evaluated on Day 11 (at the end of Dexamethasone treatment), Day 14 and Day 28.

Septic shock and acute kidney injury were defined according to previously reported data [19, 20]. Nosocomial infections (ventilator-associated pneumonia [VAP] and urinary tract infections [UTI]) were defined as bacteraemic when the microbiological diagnosis coincided with the same isolation in at least one blood culture in the absence of other specified source of bacteraemia [21].

The primary end-point was 28-day intubation rate. Secondary outcomes were Day 3-5-7 PaO_2_/FiO_2_ ratio, end-of-treatment clinical cure, septic shock, acute kidney injury, tracheostomy rate, ICU and hospital length of stay, 28-day and 90-day mortality. Safety and adverse events (AE) were determined through the biochemical abnormalities documented in medical records according to the Department of Health and Human Services—Common Terminology Criteria for Adverse Events (DHHS-CTCAE version 3.0) classification. The severity of AE was graded from 1 to 5.

### Microbiology laboratory testing

Nasopharyngeal swabs were obtained from COVID-19 patients to detect one or more SARS-CoV-2 specific nucleic acid targets by the Korean Ministry of Food and Drug Safety approved Allplex^™^ 2019-nCoV assay (Arrow Diagnostics S.r.l., Genova, Italy), which is a real-time reverse-transcriptase–polymerase-chain-reaction (RT-PCR) based assay for SARS-CoV-2 RNA detection [22]. A positive RT-PCR result was used to confirm COVID-19 diagnosis, which in turn relied on the presence of fever and/or lower respiratory tract symptoms and on lung imaging features consistent with SARS-CoV-2 pneumonia [23]. To diagnose VAP, BAL fluids or endotracheal aspirates (ET) were obtained and samples were considered positive using a threshold of ≥10^4^ (BAL) or ≥10^5^ (ET) colony forming units.

### Statistical analysis

The Kolmogorov–Smirnov test was used to evaluate the distribution of variables. Data with a non-normal distribution were assessed with the Mann–Whitney test, and the median and selected centile (25th–75th) values are given. The data with a normal distribution were assessed with the Student’s t test. Categorical variables are given as proportions, and were analysed with the chi-square test or Fisher’s exact test, as appropriate. p < 0.05 was considered significant. We included all variables in the multivariable logistic regression if they reached p ≤ 0.1 on univariate analysis. A stepwise selection procedure was used to select variables for inclusion in the final model. The Hosmer–Lemeshow goodness-of-fit test and receiver operating characteristic (ROC) curve analysis were used to assess the goodness of the logistic final model. The Kaplan–Meier method was used for the survival analysis. All statistical analyses were performed using MedCalc Statistical Software version 16.4.3 (MedCalc, Ostend, Belgium), whereas data were graphed using GraphPad Prism version 6.0 (GraphPad Software, San Diego, CA).

## Results

### Baseline characteristics and ICU presenting features of the included patients

**Fig 1:**
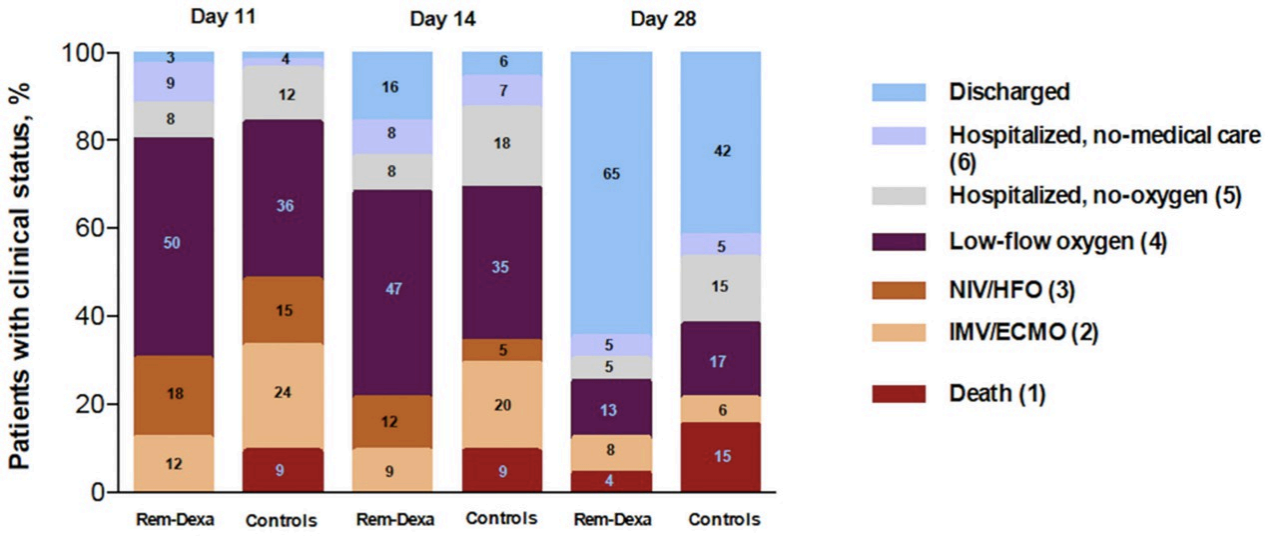
Oxygen support on Day 11, 14 and 28 in patients treated with Remdesevir-Dexamethasone and during first wave. *Rem*: Remdesevir; *Dexa*: Dexamethasone; *NIV*: non-invasive ventilation; *HFO*: high-flow oxygenation; *IMV*: invasive mechanical ventilation; *ECMO*: extracorporealmembrane oxygenation. Oxygen-support status is derived from the clinical status according to the seven-point ordinal scale, as follows: 1, death; 2, receiving invasive mechanical ventilation; 3, receiving high-flow oxygen or non-invasive ventilation; 4, receiving low-flow oxygen; 5 or 6, breathing ambient air; and 7, discharge.

During the study period 494 patients with COVID-19 induced acute respiratory failure (164 during the first wave and 330 during the second wave) were admitted to our ICUs and 292 underwent a trial of HFO/NIV. Among them 160 were excluded because received HFO/NIV <48 hours, did not receive a full course of Dexamethasone-Remdesivir, had missing data or were largely different in terms of SAPSII, SOFA, CCI scores (>5/2/1, respectively). Indeed 132 patients were included in the final analysis (66 during first wave and 66 during second wave, treated with Remdesivir+Dexamethasone (Fig 1 and Table 1). There were no significant between-cohort differences in terms of demographics and main comorbidities, apart from higher prevalence of arterial hypertension in the study group (47/66 [71.2%] vs. 34/66 [54.5%]; *p* = 0.03). Among ICU presenting features, the PaO2/FiO2 ratio was lower among Rem-Dexa group (172 [148–205] vs. 152 [126–180] mmHg; *p* = 0.01), while symptoms duration and hospital LOS were higher in patients not receiving such combination (7 [5–9] vs. 9 [7–11] days; *p*<0.01 and 3 [1–5] vs. 2 [1–3] days; *p* = 0.03). All patients during the first wave received highly-active antiretroviral therapy (darunavir or lopinavir plus ritonavir) plus hydroxychloroquine, 46 (69,7%) received IL-6 inhibitors (28 Tocilizumab and 18 Sarilumab) and 16 (24.2%) received steroids (≥0.5 mg/kg of methylprednisolone, or equivalent, for at least 5 days). Again, patients receiving Rem-Dexa had lower baseline level of main serum inflammatory markers: C-reactive protein (155.5 [71.0–195.6] vs. 87.2 [40.7–138.0] mg/dl; *p* = 0.01), procalcitonin (0.33 [0.10–5.00] vs. 0.09 [0.05–0.18] ng/ml; *p*<0.01), D-dimers (1728 [772–3714] vs. 573 [370–1109] ng/ml; *p*<0.01) and serum lactic dehydrogenase (405 [315–486] vs. 338 [283–425] mg/dl; *p* = 0.03). Conversely, patients treated with Rem-Dexa had higher white blood cells (6310 [4870–9220] vs. 9110 [7170–11802] /mm^3^; *p*<0.01) and neutrophils (5750 [3790–8142] vs. 7980 [5962–10362]/mm^3^; *p*<0.01) count. Patients of the first wave received more empirical antibiotic therapy (49/66 [74.2%] vs. 15/66 [22.7%]; *p*<0.01), with the exception of azithromycin which was similarly used (68.2% in both groups; *p* = 1).

**Table 1:** Demographic and clinical characteristics of the 132 COVID-19 patients included in the study.

Variables	Total cohort (n = 132)	First Wave (n = 66)	Rem-Dexa group (n = 66)	*p* value
*Demographics and comorbidities*				
Age (years)	64 [57–72]	67 [57–72]	64 [57–72]	0.7
Male sex, N (%)	101 (76.5)	51 (77.3)	50 (75.8)	1
Caucasian race, N (%)	121 (91.7)	60 (90.9)	61 (92.4)	1
Obesity, N (%)	36 (27.3)	20 (30.3)	16 (24.2)	0.56
Hypertension, N (%)	81 (61.4)	34 (54.5)	47 (71.2)	**0.03**
Chronic heart failure, N (%)	29 (22.0)	12 (18.2)	17 (25.8)	0.4
COPD, N (%)	17 (12.9)	7 (10.6)	10 (15.2)	0.6
Diabetes, N (%)	30 (22.7)	16 (24.2)	14 (21.2)	0.84
Neoplasm, N (%)	3 (2.3)	3 (4.5)	0 (0)	0.24
Cerebrovascular disease, N (%)	3 (2.3)	0 (0)	3 (4.5)	0.24
Chronic liver failure, N (%)	1 (0.8)	0 (0)	1 (1.5)	1
Chronic renal failure, N (%)	8 (6.1)	3 (4.5)	5 (7.6)	0.72
Immunosuppression, N (%)	5 (3.8)	2 (3.0)	3 (4.5)	1
*ICU presenting features*				
SAPS II score	30 [24–36]	31 [24–36]	29 [24–34]	0.45
SOFA score	3 [3–3]	3 [3–3]	3 [3–3]	1
CCI	2 [1–4]	3 [1–4]	2 [1–4]	0.85
Symptoms duration pre-ICU, days	8 [6–10]	7 [5–9]	9 [7–11]	**<0.01**
Hospital LOS pre-ICU, days	2 [1–4]	3 [1–5]	2 [1–3]	**0.03**
PaO_2_/FiO_2_ ratio, mmHg	160 [134–200]	172 [148–205]	152 [126–180]	**0.01**
Septic shock, N (%)	2 (1.5)	2 (3.0)	0 (0)	0.5
*Inflammatory markers*				
CRP, mg/dL	116.2 [44.5–182.1]	155.5 [71.0–195.6]	87.2 [40.7–138.0]	**0.01**
Neutrophils/mm^3^	6895 [4640–9280]	5750 [3790–8142]	7980 [5962–10362]	**<0.01**
Lymphocytes/mm^3^	775 [495–943]	760 [643–1092]	790 [590–1088]	0.54
Platelets x 10^6^/mm^3^	250 [196–337]	250.5 [196–317]	244 [197–348]	0.75
Fibrinogen, mg/dL	580 [486–693]	572 [434–734]	588 [520–764]	0.12
LDH, UI/L	376 [301–458]	405 [315–486]	338 [283–425]	**0.03**
*Concomitant medications*				
Empirical antibiotics, N (%)	64 (48.5)	49 (74.2)	15 (22.7)	**<0.01**
Azithromycin, N (%)	90 (68.2)	45 (68.2)	45 (68.2)	1
*Main clinical outcomes*				
Day 3 PaO_2_/FiO_2_ ratio, mmHg	165 [133.5–210]	176.5 [142–214]	158 [130–200]	0.1
Day 5 PaO_2_/FiO_2_ ratio, mmHg	166 [142.7–210.8]	165 (147.5–201.5)	173 (139.5–215.8)	0.67
Day 7 PaO_2_/FiO_2_ ratio, mmHg	178 [138.5–214]	171 [132–214]	186 [157–214]	0.2
EOT Clinical improvement, N(%) #	80 (60.6)	34 (51.5)	46 (69.7)	**0.05**
Endotracheal intubation, N (%)	45 (34.1)	32 (48.5)	13 (19.7)	**<0.01**
Tracheostomy, N (%)	10 (7.6)	6 (9.1)	4 (6.1)	0.75
ICU LOS, days	10 [6–17]	9 [6–16]	10 [7–18]	0.32
Hospital LOS, days	23 [16–34]	26 [17–39]	21 [16–29]	0.2
28-day mortality	13 (9.8)	10 (15.2)	3 (4.5)	0.08
90-day mortality	18 (13.6)	11 (16.7)	7 (10.6)	0.45

Data are presented as median [IQR], unless otherwise indicated

*SOC*: standard of care; *Rem*: Remdesevir; *Dexa*: Dexamethasone; *COPD*: chronic obstructive pulmonary disease; *ICU*: intensive care unit; *SAPS II*: Simplified Acute Physiology Score; *SOFA*: Sequential Organ Failure Assessment; *CCI*: Charlson Comorbidity Index; *CRP*: C-reactive protein; *WBC*: white blood cells; *LDH*: lactate dehydrogenase; *NIV*: non-invasive ventilation; *HFO*: high-flow nasal cannula oxygenation; *LOS*: length of stay; *IQR*: interquartile range; *ICU*: intensive care unit; *PaO*_*2*_*/FiO*_*2*_: ratio of partial pressure of arterial oxygen to fraction of inspired oxygen; *EOT*: end of treatment.

* Within 48 hours from ICU admission

^#^Defined as an improvement of at least 1 point from the baseline score of 3 (high-flow oxygenation or non-invasive ventilation).

### Outcomes and predictors of endotracheal intubation

The 28-day endotracheal intubation rate was significantly lower in patients receiving Rem-Dexa (32/66 [48.5%] vs. 13/66 [19.7%]; *p*<0.01) (Table 1). Moreover, the study group patients showed higher rate of end-of-treatment clinical improvement (34/66 [51.5%] vs. 46/66 [69.7%]; *p* = 0.05) (Fig 1). On day 11 after ICU admission, a similar proportion of patients were still receiving HFO/NIV (15% vs. 18%; *p* = 0.81), 70% of Rem-Dexa patients and 52% during first wave (*p* = 0.05) improved their clinical status while only 12% of Rem-Dexa patients (*p* = 0.01) were on invasive mechanical ventilation/extracorporeal membrane oxygenation or died. At the end of the observation, no patient was still receiving HFO/NIV and the 28-day hospital discharge rate was 65% in Rem-Dexa group compared with 42% in those ones not receiving such combination (p = 0.01). PaO2/FiO2 ratio of Rem-Dexa group and first wave patients was not statistically different at each time-point (Table 1).

The ICU and hospital length of stay was similar between groups (*p* = 0.32 and *p* = 0.2, respectively). Despite being lower among patients receiving Rem-Dexa, the 28-day and 90-day mortality rate was not statistically different (*p* = 0.08 and *p* = 0.45, respectively).

**Fig 2:**
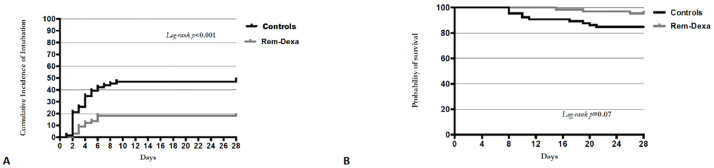
Kaplan-Meier curve showing the impact of Remdesevir-Dexamethasone treatment (grey line) on 28-day intubation rate and mortality. **A**: Kaplan-Meier curve showing the impact of Remdesevir-Dexamethasone treatment (grey line) on 28-day intubation rate. The multivariate Cox-regression model showed 28-day ETI rate to be reduced with Remdesevir-Dexamethasone (Rem-Dexa) treatment (HR 0.31, 95% CI 0.16–0.61), and the difference in ETI rate between treatment groups was also found on Kaplan–Meier survival curve analysis (p< 0.001). **B**: Kaplan-Meier curve showing the impact of Remdesevir-Dexamethasone treatment (grey line) on 28-day mortality. The multivariate Cox-regression model did not show 28-day survival rate to be reduced with Remdesevir-Dexamethasone (Rem-Dexa) treatment (HR 0.33, 95% CI 0.09–1.18), and no significant difference in 28-day mortality rate between treatment groups was found on Kaplan–Meier survival curve analysis (p< 0.07).

Among the overall population, 45 patients underwent endotracheal intubation (ETI) after HFO/NIV failure (Table 1). The univariate analysis (Table 2) showed that higher SAPS II (*p*<0.01), SOFA score (*p* = 0.02), serum C-reactive protein (*p*<0.01), procalcitonin (*p* = 0.02) and lactic dehydrogenase (*p* = 0.01) as well as lower PaO2/FiO2 ratio (*p* = 0.03) and lymphocytes count (*p* = 0.04) were associated with higher risk of 28-day ETI. In addition, treatment with Rem-Dexa was more likely to occur in patients who were not intubated (*p*<0.01), while the administration of empirical antibiotics was associated with ETI (*p* = 0.01). However, the multivariate logistic regression analysis showed that higher SAPS II (adjusted odds ratio [OR], 1.09; 95% confidence interval [CI], 1.02–1.18; *p* = 0.02) and serum lactic dehydrogenase (adjusted OR, 1.01; 96% CI, 1.00–1.01; *p* = 0.01) as well as lower PaO2/FiO2 ratio (adjusted OR, 0.98; 95% CI, 0.97–0.99; *p* = 0.04) were independently associated with endotracheal intubation. Conversely, treatment with Rem-Dexa was independently associated with a reduced risk of ETI (adjusted OR, 0.22; 95% CI, 0.05–0.94; *p* = 0.04). The multivariate Cox-regression model confirmed that patients of the study group had a significantly reduced risk of intubation (hazard ratio [HR], 0.31; 95% CI, 0.16–0.61), consistently with the Kaplan–Meier curve analysis (*p*<0.01) illustrated in Fig 2A. Again, the Cox-regression model showed that treatment with Rem-Dexa did not significantly improve the 28-day survival, as confirmed by the Kaplan-Meier curve analysis (*p* = 0.07) presented in Fig 2B.

**Table 2:** Univariate and multivariate analysis of factors associated with ETI.

Variables	No. (%) of patients		Univariate analysis		Adjusted analysis	
	ETI + (*n* = 45)	ETI—(*n* = 87)	*P* value	OR (95% CI)	*P* value	OR (95% CI)
*Demographics and comorbidities*						
Age (years)	66 [60–71]	63 [53–73]	0.12	1.02 (0.99–1.06)	-	-
Male sex, N (%)	38 (84.4)	63 (72.4)	0.13	2.07 (0.81–5.26)	**-**	**-**
Caucasian race, N (%)	44 (97.8)	77 (88.5)	0.1	5.7 (0.71–46.1)	-	-
Obesity, N (%)	12 (26.7)	24 (27.6)	0.91	0.95 (0.42–2.15)	**-**	**-**
Hypertension, N (%)	30 (66.7)	51 (58.6)	0.37	1.41 (0.67–2.99)	**-**	**-**
Chronic heart failure, N (%)	11 (24.4)	18 (20.7)	0.62	1.24 (0.53–2.92)	**-**	**-**
COPD, N (%)	6 (13.3)	11 (12.6)	0.91	1.06 (0.37–3.09)	**-**	**-**
Diabetes, N (%)	14 (31.1)	16 (18.4)	0.11	2.00 (0.87–4.61)	**-**	**-**
Neoplasm, N (%)	2 (4.4)	1 (1.1)	0.25	4.00 (0.35–45.4)	**-**	**-**
Cerebrovascular disease, N (%)	0 (0)	3 (3.4)	0.99	0 (-)	**-**	**-**
Chronic liver failure, N (%)	0 (0)	1 (1.1)	0.99	0 (-)	**-**	**-**
Chronic renal failure, N (%)	2 (4.4)	6 (6.9)	0.58	0.63 (0.12–3.25)	**-**	**-**
Immunosuppression, N (%)	1 (2.2)	4 (4.6)	0.51	0.47 (0.05–4.35)	**-**	**-**
*ICU presenting features*						
**SAPS II score**	**34 [28–41]**	**28 [22–34]**	**<0.01**	**1.09 (1.04–1.15)**	**0.02**	**1.09 (1.02–1.18)**
SOFA score	3 [3–4]	3 [3–3]	0.02	1.66 (1.09–2.53)	0.09	1.77 (0.92–3.42)
CCI	3 [2–4]	2 [1–4]	0.12	1.18 (0.96–1.44)	**-**	**-**
**PaO**_**2**_**/FiO**_**2**_ **ratio, mmHg**	**151 [128–181]**	**168 [139–211]**	**0.03**	**0.99 (0.98–0.99)**	**0.04**	**0.98 (0.97–0.99)**
Septic shock, N (%)	1 (2.2)	1 (1.1)	0.64	1.95 (0.12–31.00)	**-**	**-**
CRP, mg/dL	158.5 [65.5–229.0]	103.4 [42.5–160.4]	<0.01	1.010 (1.002–1.011)	0.38	1.01 (0.99–1.01)
Neutrophils/mm^3^	6205 [4485–9055]	7535 [4700–9440]	0.08	0.99 (0.99–1.00)	-	-
Lymphocytes/mm^3^	670 [515–985]	815 [600–1200]	0.04	0.99 (0.99–1.00)	0.1	0.99 (0.99–1.01)
**LDH, UI/L**	**424 [340–506]**	**344 [288–419]**	**0.01**	**1.003 (1.001–1.007)**	**0.01**	**1.007 (1.002–1.010)**
*Concomitant medications*						
**Rem-Dexa, N (%)**	**13 (28.9)**	**53 (60.9)**	**<0.01**	**0.26 (0.12–0.57)**	**0.04**	**0.22 (0.05–0.94)**
Empirical antibiotics, N (%)	30 (66.7)	34 (39.1)	0.01	3.10 (1.46–6.63)	0.25	2.10 (0.60–7.12)
Azithromycin, N (%)	30 (66.7)	60 (69.0)	0.79	0.90 (0.42–1.95)	**-**	**-**

We included all variables in the multivariable logistic regression if they reached p ≤ 0.05 on univariate analysis. A stepwise selection procedure was used to select variables for inclusion in the final model. ROC curve analysis was used to assess the goodness of the final logistic regression model (AUC ± SE = 0.89±0.03 with 95%CI 0.81–0.94; chi-square statistics p < 0.001)

Data are presented as median [IQR], unless otherwise indicated

*Rem*: Remdesevir; *Dexa*: Dexamethasone; *ETI*: endotracheal intubation; *OR*: odds ratio; *CI*: confidence interval; *COPD*: chronic obstructive pulmonary disease; *ICU*: intensive care unit; *SAPS II*: Simplified Acute Physiology Score; *SOFA*: Sequential Organ Failure Assessment; *CCI*: Charlson Comorbidity Index; *CRP*: C-reactive protein; *LDH*: lactate dehydrogenase; *Dexa*: Dexamethasone; *ROC*: receiver operating characteristic; *AUC*: area under the curve; *SE*: standard error; *IQR*: interquartile range.

### Adverse events

In the overall study population, the most frequent abnormal laboratory measures within the first 10 days after treatment initiation (Table 3) were increase of aspartate aminotransferase (37.9%), anaemia (29.5%), acute renal failure with Kidney Disease Improving Global Guidelines stage > 2 (15.9%) and thrombocytopenia (15.2%). Anaemia and increase of alanine aminotransferase were significantly higher in patients not treated with Rem-Dexa (26/66 [39.4%] vs. 13/66 [19.7%]; *p* = 0.02 and 9/66 [13.6%] vs. 1/66 [1.5%]; *p* = 0.02, respectively). In the overall population, among potentially associated clinical complications, nosocomial infections (31.1%) and insulin use for hyperglycaemia (26.5%) were the most frequently observed, occurring with similar rate in the two groups.

**Table 3:** Comparison of adverse events between the two groups.

Variables	Total cohort (n = 132)	First Wave (n = 66)	Rem-Dexa group (n = 66)	*p* value
*Abnormal laboratory measures within the first 10 days after treatment initiation*				
Neutropenia, N (%)	5 (3.8)	5 (7.6)	0 (0)	0.06
**Anemia, N (%)**	**39 (29.5)**	**26 (39.4)**	**13 (19.7)**	**0.02**
Thrombocytopenia, N (%)	20 (15.2)	11 (16.7)	9 (13.6)	0.81
AST > 5 N, N (%)	5 (37.9)	5 (7.6)	0 (0)	0.06
**ALT> 5 N, N (%)**	**10 (7.6)**	**9 (13.6)**	**1 (1.5)**	**0.02**
ARF KDIGO ≥2, N (%)	21 (15.9)	12 (18.2)	9 (13.6)	0.64
CRRT, N (%)	10 (7.6)	4 (6.1)	6 (9.1)	0.75
QT interval > 450 ms, N (%)	5 (3.8)	5 (7.6)	0 (0)	0.06
*Potentially associated clinical complications*				
Insulin use for hyperglycemia, N (%)	35 (26.5)	15 (22.7)	20 (30.3)	0.43
ICU nosocomial infection N (%)	41 (31.1)	20 (30.3)	21 (31.8)	1
VAP, N (%)	19 (14.4)	11 (16.7)	8 (12.1)	0.62
UTI, N (%)	21 (15.9)	8 (12.1)	13 (19.7)	0.34
BSI, N (%)	13 (9.8)	6 (9.1)	7 (10.6)	1

Data are presented as absolute value (%)

*SOC*: standard of care; *Rem*: Remdesevir; *Dexa*: Dexamethasone; *AST*: aspartate aminotransferase: *ALT*: alanine aminotransferase; *ARF*: acute renal failure; *KDIGO*: Kidney Disease Improving Global Guidelines; *CRRT*: continuous renal replacement therapy; *ICU*: intensive care unit; *VAP*: ventilator-associated pneumonia; *UTI*: urinary tract infection; *BSI*: bloodstream infection.

## Discussion

We studied the concomitant Remdesivir and Dexamethasone therapy in COVID-19 critically ill patients who needed HFOT or NIV, a population where the efficacy of this association is still a matter of debate [24]. Early Rem-Dexa administration significantly reduced 28-day intubation rate, increasing the percentage of clinical improvement at the end of treatment. This approach was not associated with more laboratory neither clinical adverse events.

The use of Remdesivir for SARS-COV-2 associated respiratory failure displays a strong biological rationale. The activity of this molecule activity against *Coronaviridae* consists in the inhibition of viral RNA-dependent RNA-polymerase [25, 26], providing clinical benefits in animal pneumonia models [11, 27, 28]. Currently Remdesivir is the only Food and Drug Administration approved molecule for COVID-19 and National Institute of Health (NIH) guidelines recommend its use in patients requiring minimal oxygen supplement (BIIa) [15]. However in those who are more severe and need HFOT or NIV, its adoption has not been rigorously studied (BIII). The strongest data supporting the use of Remdesivir derives from the Adaptive COVID-19 Treatment Trial (ACTT-1), where it reduced the median time to recovery from 15 days (placebo) to 10 days (RR 1.29; 95% CI 1.12–1.49), albeit in the subgroup of patients receiving HFOT/NIV (193) this advantage was not observed [12].

In our study, the two populations were homogeneous in terms of clinical presentation and severity (SAPSII, SOFA scores and CCI), the respiratory management (HFOT and NIV use) was well standardized [29] and the treatment duration, in line with *Goldman et al*. results, was 5 days and completed after endotracheal intubation [13]. Then we can suppose that also in such border-line patients category, mainly treated within 10 days from symptoms onset and immediately after ICU admission, Remdesivir could have reduced lung tissue damage, preventing further respiratory deterioration.

Our results are actually in line with smaller investigations in this setting: in 51 mechanically ventilated patients, *Pasquini et al*. observed that Remdesivir administration was significantly associated with improved survival [30] similarly, during first pandemic surge in Lombardy (Italy), in a cohort of 113 intubated patients, those who received the antiviral treatment experienced lower mortality (15.2% vs. 38.8%), higher rates of extubation (88% vs. 60%), median ventilator-free days (11 vs. 5) and hospital discharge (85% vs. 59%) [31]. Again, in a post-hoc analysis of two randomized controlled trials, the 312 patients treated with Remdesivir (21% on mechanical ventilation) showed higher recovery (74.4% vs.59%) and survival (92.4% vs. 87.5%) rates, compared with standard of care [32].

Dexamethasone represents the mainstay of severe COVID-19 pharmacological treatment. NIH experts Panel recommend its use at a dosage of 6 mg h/24 for 10 days in patients requiring rapidly increasing amount of oxygen, HFOT, NIV and invasive mechanical ventilation (AI) [15].The biological rational of steroids use in this disease stands in the strong anti-inflammatory response associated with SARS-COV 2 infections which appears to blunt the overwhelming innate and adaptive body response, rapidly damaging lung tissue [33]. In the RECOVERY trial, 4321 patients receiving supplemental oxygen at enrolment and treated with Dexamethasone at a dose of 6 mg once daily up to 10 days, showed a significant survival benefit compared with usual care (death rate 23.3% vs. 26.2%; RR 0.82; 95%CI: 0.72–0.94) [8]. Although, we do not have conclusive data in the subpopulation on HFOT/NIV, we can suppose that more severe is patients’ respiratory impairment, more benefit may derive from dexamethasone administration.

In our study, almost all subjects in the first wave received anti-inflammatory treatments with other steroids or IL-6 inhibitors, but the absence of a protocoled administration in terms of dosages and duration may have reduced the potential beneficial effects. In addition, in Rem-Dexa group, patients started Dexamethasone in the Emergency Department, potentially explaining the ICU presenting lower inflammatory burden. Due to the lack of rigorous clinical trials, the safety and efficacy of Remdesivir plus Dexamethasone combination is still unknown. The biological rationale of such combination is strong: severe COVID-19 is characterized by a multiple organ failure, mainly driven by an overwhelming inflammatory response, which may be dampened by steroids administration. Conversely, the use of Remdesivir may counteract the decrease in viral clearance, potentially promoted by Dexamethasone [34]. The benefits of Rem-Dexa observed in our study are in line with a recent large Danish observational investigation [35], where, including a less severe cohort (3% were ventilated within the first 24 hours), the above combination was associated with a 6.9% absolute reduction in 30-day mortality, corresponding to a weighted OR of 0.47 (95%CI, 0.38–0.57).

All these data do not really permit to identify the individual effect of each of the two drugs on the observed outcomes, but in our study we can speculate a clinical plus-value of Remdesivir. Almost all patients during the first wave were treated with an anti-inflammatory agent (steroids or IL-6 inhibitors) and patients in the Rem-Dexa group received Remdesevir at ICU admission, after initial clinical impairment, despite Dexamethasone initiation in the emergency department (1 or 2 days earlier).

This study has some limitations. First, its monocentric (two ICUs) design could limit the applicability of our findings to other centres. Second, the sample size was relatively small and the nature of the study is purely observational. Third, we did not monitor the course of neither inflammation or viral replication after treatment initiation, to evaluate the biochemical and microbiological response. Indeed, we cannot quantify the single impact of either Remdesivir or Dexamethasone on the observed clinical outcomes.

## Conclusions

This is the first study where the clinical effect of Remdesivir and Dexamethasone combination has been investigated in COVID-19 critically ill patients needing HFOT or NIV. We observed that early adoption of such combination was associated with lower intubation rate and better clinical outcome at the end of treatment, without additional adverse effects. These results may represent a proof-of-concept for future interventional study.

## References

[1] Chang D, Lin M, Wei L, Xie L, Zhu G, Cruz CD, et al. Epidemiologic and Clinical Characteristics of Novel Coronavirus Infections Involving 13 Patients Outside Wuhan, China. JAMA. 2020;323(11):1092–3. doi: 10.1001/jama.2020.1623 32031568 PMC7042871

[2] Grasselli G, Zangrillo A, Zanella A, Antonelli M, Cabrini L, Castelli A, et al. Baseline Characteristics and Outcomes of 1591 Patients Infected With SARS-CoV-2 Admitted to ICUs of the Lombardy Region, Italy. JAMA. 2020;323(16):1574–81. doi: 10.1001/jama.2020.5394 32250385 PMC7136855

[3] Grieco D, Menga L, Cesarano M, Rosà T, Spadaro S, Bitondo M, et al. Effect of Helmet Noninvasive Ventilation vs High-Flow Nasal Oxygen on Days Free of Respiratory Support in Patients With COVID-19 and Moderate to Severe Hypoxemic Respiratory Failure: The HENIVOT Randomized Clinical Trial. JAMA. 2021;325(17):1731–43. doi: 10.1001/jama.2021.4682 33764378 PMC7995134

[4] Rochwerg B, Einav S, Chaudhuri D, Mancebo J, Mauri T, Helviz Y, et al. The role for high flow nasal cannula as a respiratory support strategy in adults: a clinical practice guideline. Intensive Care Med. 2020;46(12):2226–37. doi: 10.1007/s00134-020-06312-y 33201321 PMC7670292

[5] Steinberg K, Hudson L, Goodman R, Hough C, Lanken P, Hyzy R, et al. Efficacy and safety of corticosteroids for persistent acute respiratory distress syndrome. N Engl J Med. 2006;354(16):1671–84. doi: 10.1056/NEJMoa051693 16625008

[6] Rhen T, Cidlowski J. Antiinflammatory action of glucocorticoids—new mechanisms for old drugs. N Engl J Med. 2005;353(16):1711–23. doi: 10.1056/NEJMra050541 16236742

[7] Villar J, Ferrando C, Martínez D, Ambrós A, Muñoz T, Soler J, et al. Dexamethasone treatment for the acute respiratory distress syndrome: a multicentre, randomised controlled trial. Lancet Respir Med. 2020;8(3):267–76. doi: 10.1016/S2213-2600(19)30417-5 32043986

[8] RECOVERY Collaborative Group, Horby P, Lim W, Emberson J, Mafham M, Bell J, et al. Dexamethasone in Hospitalized Patients with Covid-19. N Engl J Med. 2021;384(8):693–704. doi: 10.1056/NEJMoa2021436 32678530 PMC7383595

[9] Cao B, Wang Y, Wen D, Liu W, Wang J, Fan G, et al. A Trial of Lopinavir-Ritonavir in Adults Hospitalized with Severe Covid-19. N Engl J Med. 2020;382(19):1787–99. doi: 10.1056/NEJMoa2001282 32187464 PMC7121492

[10] Borba M, Val F, Sampaio V, Alexandre M, Melo G, Brito M, et al. Effect of High vs Low Doses of Chloroquine Diphosphate as Adjunctive Therapy for Patients Hospitalized With Severe Acute Respiratory Syndrome Coronavirus 2 (SARS-CoV-2) Infection: A Randomized Clinical Trial. JAMA Netw Open. 2020;3(4):e208857. doi: 10.1001/jamanetworkopen.2020.8857 32330277 PMC12124691

[11] Wang M, Cao R, Zhang L, Yang X, Liu J, Xu M, et al. Remdesivir and chloroquine effectively inhibit the recently emerged novel coronavirus (2019-nCoV) in vitro. Cell Res. 2020;30(3):269–71. doi: 10.1038/s41422-020-0282-0 32020029 PMC7054408

[12] Beigel J, Tomashek K, Dodd L, Mehta A, Zingman B, Kalil A, et al. Remdesivir for the Treatment of Covid-19—Final Report. N Engl J Med. 2020;383(19):1813–26. doi: 10.1056/NEJMoa2007764 32445440 PMC7262788

[13] Goldman J, Lye D, Hui D, Marks K, Bruno R, Montejano R, et al. Remdesivir for 5 or 10 Days in Patients with Severe Covid-19. N Engl J Med. 2020;383(19):1827–37. doi: 10.1056/NEJMoa2015301 32459919 PMC7377062

[14] Cutuli S, Carelli S, Grieco D, De Pascale G. Immune Modulation in Critically Ill Septic Patients. Medicina (Kaunas). 2021;57(6):552. doi: 10.3390/medicina57060552 34072649 PMC8226671

[15] NIH. Coronavirus Disease 2019 (COVID-19) Treatment Guidelines 2020 [https://COVID-19treatmentguidelines.nih.gov/. Nih 2019:130.

[16] Le Gall J, Lemeshow S, Saulnier F. A new Simplified Acute Physiology Score (SAPS II) based on a European/North American multicenter study. JAMA. 1993;270(24):2957–63. doi: 10.1001/jama.270.24.2957 8254858

[17] Vincent J, Moreno R, Takala J, Willatts S, Mendonça AD, Bruining H, et al. The SOFA (Sepsis-related Organ Failure Assessment) score to describe organ dysfunction/failure. On behalf of the Working Group on Sepsis-Related Problems of the European Society of Intensive Care Medicine. Intensive Care Med. 1996;22(7):707–10. doi: 10.1007/BF01709751 8844239

[18] Charlson M, Pompei P, Ales K, MacKenzie C. A new method of classifying prognostic comorbidity in longitudinal studies: development and validation. J Chronic Dis. 1987;40(5):373–83. doi: 10.1016/0021-9681(87)90171-8 3558716

[19] Rhodes A, Evans L, Alhazzani W, Levy M, Antonelli M, Ferrer R, et al. Surviving Sepsis Campaign: International Guidelines for Management of Sepsis and Septic Shock: 2016. Intensive Care Med. 2017;43(3):304–77. doi: 10.1007/s00134-017-4683-6 28101605

[20] KDIGO AKI Writing Group. Kidney Disease: Improving Global Outcomes (KDIGO) clinical practice guideline for acute kidney injury. Kidney Int. 2012;2(Suppl 1):1–141.

[21] De la Calle C, Morata L, Cobos-Trigueros N, Martinez J, Cardozo C, Mensa J, et al. Staphylococcus aureus bacteremic pneumonia. Eur J Clin Microbiol Infect Dis. 2016;35(3):497–502. doi: 10.1007/s10096-015-2566-8 26780692

[22] Hanson K, Caliendo A, Arias C, Hayden M, Englund J, Lee M, et al. The Infectious Diseases Society of America Guidelines on the Diagnosis of COVID-19: Molecular Diagnostic Testing. Clin Infect Dis. 2021;Online ahead of print. 10.1093/cid/ciab048 PMC7929045 33480973

[23] Wiersinga W, Rhodes A, Cheng A, Peacock S, Prescott H. Pathophysiology, Transmission, Diagnosis, and Treatment of Coronavirus Disease 2019 (COVID-19): A Review. JAMA. 2020;324(8):782–93. doi: 10.1001/jama.2020.12839 32648899

[24] Beigel J. What is the role of remdesivir in patients with COVID-19? Curr Opin Crit Care. 2021;Online ahead of print. doi: 10.1097/MCC.0000000000000866 34353998 PMC8416929

[25] Nili A, Farbod A, Neishabouri A, Mozafarihashjin M, Tavakolpour S, Mahmoudi H. Remdesivir: A beacon of hope from Ebola virus disease to COVID-19. Rev Med Virol. 2020;30(6):1–13. doi: 10.1002/rmv.2133 33210457

[26] Borbone N, Piccialli G, Roviello G, Oliviero G. Nucleoside Analogs and Nucleoside Precursors as Drugs in the Fight against SARS-CoV-2 and Other Coronaviruses. Molecules. 2021;26(4):986. doi: 10.3390/molecules26040986 33668428 PMC7918729

[27] Sheahan T, Sims A, Graham R, Menachery V, Gralinski L, Case J, et al. Broad-spectrum antiviral GS-5734 inhibits both epidemic and zoonotic coronaviruses. Sci Transl Med. 2017;9(396):eaal3653. doi: 10.1126/scitranslmed.aal3653 28659436 PMC5567817

[28] Williamson B, Feldmann F, Schwarz B, Meade-White K, Porter D, Schulz J, et al. Clinical benefit of remdesivir in rhesus macaques infected with SARS-CoV-2. Nature. 2020;585(7824):273–6. doi: 10.1038/s41586-020-2423-5 32516797 PMC7486271

[29] Grieco D, Maggiore S, Roca O, Spinelli E, Patel B, Thille A, et al. Non-invasive ventilatory support and high-flow nasal oxygen as first-line treatment of acute hypoxemic respiratory failure and ARDS. Intensive Care Med. 2021;47(8):851–66. doi: 10.1007/s00134-021-06459-2 34232336 PMC8261815

[30] Pasquini Z, Montalti R, Temperoni C, Canovari B, Mancini M, Tempesta M, et al. Effectiveness of remdesivir in patients with COVID-19 under mechanical ventilation in an Italian ICU. J Antimicrob Chemother. 2020;75(11):3359–65. doi: 10.1093/jac/dkaa321 32829390 PMC7499641

[31] Lapadula G, Bernasconi D, Bellani G, Soria A, Rona R, Bombino M, et al. Remdesivir Use in Patients Requiring Mechanical Ventilation due to COVID-19. Open Forum Infect Dis. 2020;7(11):ofaa481. doi: 10.1093/ofid/ofaa481 33204761 PMC7651598

[32] Olender S, Perez K, Go A, Balani B, Price-Haywood E, Shah N, et al. Remdesivir for Severe COVID-19 versus a Cohort Receiving Standard of Care. Clin Infect Dis. 2020;Online ahead of print. 10.1093/cid/ciaa1041 PMC7454434 32706859

[33] Arabi Y, Chrousos G, Meduri G. The ten reasons why corticosteroid therapy reduces mortality in severe COVID-19. Intensive Care Med. 2020;Online ahead of print. doi: 10.1007/s00134-020-06223-y 33026460 PMC7538533

[34] Arabi Y, Mandourah Y, Al-Hameed F, Sindi A, Almekhlafi G, Hussein MA, et al. Corticosteroid Therapy for Critically Ill Patients with Middle East Respiratory Syndrome. Am J Respir Crit Care Med. 2018;197(6):757–67. doi: 10.1164/rccm.201706-1172OC 29161116

[35] Benfield T, Bodilsen J, Brieghel C, Harboe Z, Helleberg M, Holm C, et al. Improved survival among hospitalized patients with COVID-19 treated with remdesivir and dexamethasone. A nationwide population-based cohort study. Clin Infect Dis. 2021;Online ahead of print. doi: 10.1093/cid/ciab536 34111274 PMC8344480

